# Epstein–Barr virus and multiple sclerosis: moving from questions of association to questions of mechanism

**DOI:** 10.1002/cti2.1451

**Published:** 2023-05-17

**Authors:** Olivia G Thomas, Alan Rickinson, Umaimainthan Palendira

**Affiliations:** ^1^ Department of Clinical Neuroscience, Therapeutic Immune Design, Centre for Molecular Medicine Karolinska Institute Stockholm Sweden; ^2^ Institute of Cancer and Genomic Sciences, College of Medical and Dental Sciences University of Birmingham, Edgbaston Birmingham UK; ^3^ School of Medical Sciences, Faculty of Medicine and Health The University of Sydney Camperdown NSW Australia; ^4^ Charles Perkins Centre The University of Sydney Camperdown NSW Australia

**Keywords:** autoimmunity, Epstein–Barr virus, infectious mononucleosis, molecular mimicry, multiple sclerosis, neuroinflammation

## Abstract

The link between Epstein–Barr virus (EBV) and multiple sclerosis (MS) has puzzled researchers since it was first discovered over 40 years ago. Until that point, EBV was primarily viewed as a cancer‐causing agent, but the culmination of evidence now shows that EBV has a pivotal role in development of MS. Early MS disease is characterised by episodic neuroinflammation and focal lesions in the central nervous system (CNS) that over time develop into progressive neurodegeneration and disability. Risk of MS is vanishingly low in EBV seronegative individuals, history of infectious mononucleosis (acute symptomatic primary infection with EBV) significantly increases risk and elevated antibody titres directed against EBV antigens are well‐characterised in patients. However, the underlying mechanism – or mechanisms – responsible for this interplay remains to be fully elucidated; how does EBV‐induced immune dysregulation either trigger or drive MS in susceptible individuals? Furthermore, deep understanding of virological and immunological events during primary infection and long‐term persistence in B cells will help to answer the many questions that remain regarding MS pathogenesis. This review discusses the current evidence and mechanisms surrounding EBV and MS, which have important implications for the future of MS therapies and prevention.

## Graphical abstract

The link between Epstein–Barr virus (EBV) and multiple sclerosis (MS) has puzzled researchers since it was first discovered over 40 years ago. This review discusses the current evidence and mechanisms surrounding EBV and MS, which have important implications for the future of MS therapies and prevention.

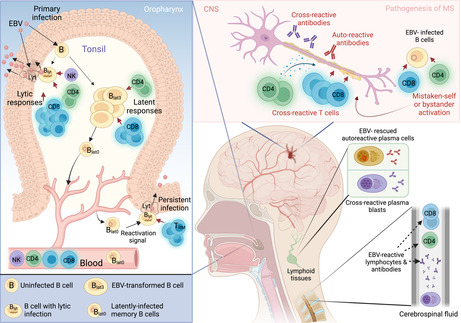

## Introduction

Epstein–Barr virus (EBV) is arguably one of the most successful pathogens to infect humans. Over 90% people in all countries naturally acquire the virus, often in their first decade, and will thereafter carry EBV for life as a latent, usually asymptomatic, infection. However, this seemingly innocuous agent has a much more sinister side to its character. It is causally linked to at least one severe infectious disease, infectious mononucleosis (IM), and, remarkably, to at least seven different types of human cancer. With regard to IM, EBV causes a self‐limiting lymphoproliferative disease (LPD) characterised by fever, lymphadenopathy, sore throat, malaise and an extensive expansion of T cells in the blood; most interestingly, IM is a relatively common accompaniment of the delayed primary infection^1^ that often occurs in adolescents and young adults in affluent societies but is seen very rarely in poorer countries where the virus is typically acquired in the first decade of life.

With regard to EBV's links to cancer, its contribution to the malignant cell phenotype differs in detail between the different tumor types, but consistently involves the expression of one or more of the latent cycle genes of the virus. Its direct action is, however, most obvious in the fatal B‐LPD, a malignancy first recognised in transplant recipients receiving T cell‐suppressive therapy, hence its original description as ‘post‐transplant lymphoma’. Here, EBV appears to be the main driver of B‐cell growth, its action recapitulating what happens when EBV infects B cells *in vitro* and, through the expression of its complete set of latent cycle genes (EBNA1, EBNA2, EBNA3A, EBNA3B, EBNA3C, EBNA‐LP, LMP1, LMP2 and noncoding RNAs including EBERs and miRNAs), transforms those cells into permanent EBV genome‐positive lymphoblastoid cell lines (LCLs). This link to LPD of the immunocompromised not only demonstrates EBV's oncogenic potential but also strongly implies that, in the immunocompetent host, T‐cell surveillance plays a critical role in maintaining the virus–host balance. For many years, research in the EBV field has largely focussed on the B‐cell growth‐transforming ability of the virus, on its role as the causative agent of IM, and on its links to an unexpectedly wide range of malignancies. However, the field is now turning its attention to the mounting evidence that this same virus is also aetiologically linked to the most common chronic inflammatory condition of the central nervous system (CNS).

Multiple sclerosis (MS) is a chronic disease of the CNS caused by a complex interplay of inflammatory and neurodegenerative mechanisms. Multiple sclerosis is most commonly diagnosed in women between 20 and 40 years of age with the highest incidence occurring in higher latitude regions such as in Western Europe and the United States.^2^ Symptoms are caused by focal lesions, which can develop anywhere in the CNS with most cases (approximately 85%) presenting a relapsing–remitting disease course, which is characterised by periods of symptom exacerbation between periods of clinical remission. Over time, relapses become less frequent and neurodegenerative mechanisms take over, causing a steady loss of brain volume and function.

Both genetic and environmental factors have been implicated in the aetiology of this complex disease. However, poor disease concordance between genetically identical twins points to a significant role for specific environmental triggers, the precise identity and sequence of which remain unknown; however, vitamin D, smoking, obesity and infections have all been implicated.^3^ An infectious aetiology was suspected almost from the time MS was first described. Jean‐Martin Charcot, a French neurologist, was the first to name and describe MS in the nineteenth century. One of his students, Pierre Marie, later postulated an infectious aetiology for MS but scientific evidence was lacking. Medical records from the Faeroe Islands and Iceland also suggested that increased incidence of MS‐like illness coincided with the arrival of the foreign troops on the islands during the Second World War and propelling the infectious aetiology theory, although later studies showed that this was likely because of a combination of other factors, such as improved diagnosis and increased familial aetiology in genetically homogeneous populations.^4^, ^5^ Over the years, many different pathogens have been implicated in the pathogenesis of MS but, in 1981, Warner and Carp were the first to suggest a role for EBV.^6^ A potential role for EBV was based on previous epidemiological observations that showed rare incidence of symptomatic infection in nonaffluent countries, whereas delayed infection was mostly seen in affluent Caucasians and was often symptomatic; both of these observations were reminiscent of the incidence and age profile of MS – a disease also more common in Western societies. More compelling evidence, however, came in 2010, when Alberto Ascherio's group showed the potential link between primary infection with EBV and MS in a large cohort study of US military personnel.^7^ More recently, as described below, the same group has further strengthened this observation by investigating in a much larger cohort of military recruits and demonstrating convincingly the role of EBV in MS.^8^

## Immunobiology of EBV infection

Epstein–Barr virus is a γ‐1 Herpesvirus with a large double‐stranded DNA genome of > 170 Kb in size and encodes > 90 viral proteins, plus a range of noncoding viral RNAs. A significant fraction of EBV's protein‐coding genes have recognisable homologues in the other human Herpesviruses and encode essential components of the Herpesvirus replication machinery. However, many of the EBV's other genes are specific to EBV, reflecting the unique lifestyle of this virus and the complexity of the virus–host relationship. For both scientific and logistic reasons, there is a paucity of authentic animal models to study this relationship in depth. Much therefore depends on studies in the natural host, in particular on primary infection in IM patients, on the virus‐carrier state in healthy individuals, on EBV infection in T‐cell‐suppressed individuals and in patients with genetically defined primary immunodeficiencies. Despite much progress in all of these areas, current understanding of EBV biology, of the nature of virus persistence and of its relationship to the host immune response, remains incomplete. This fact needs to be borne in mind throughout all discussions of EBV disease pathogenesis, particularly in the context of MS.

**Figure 1:**
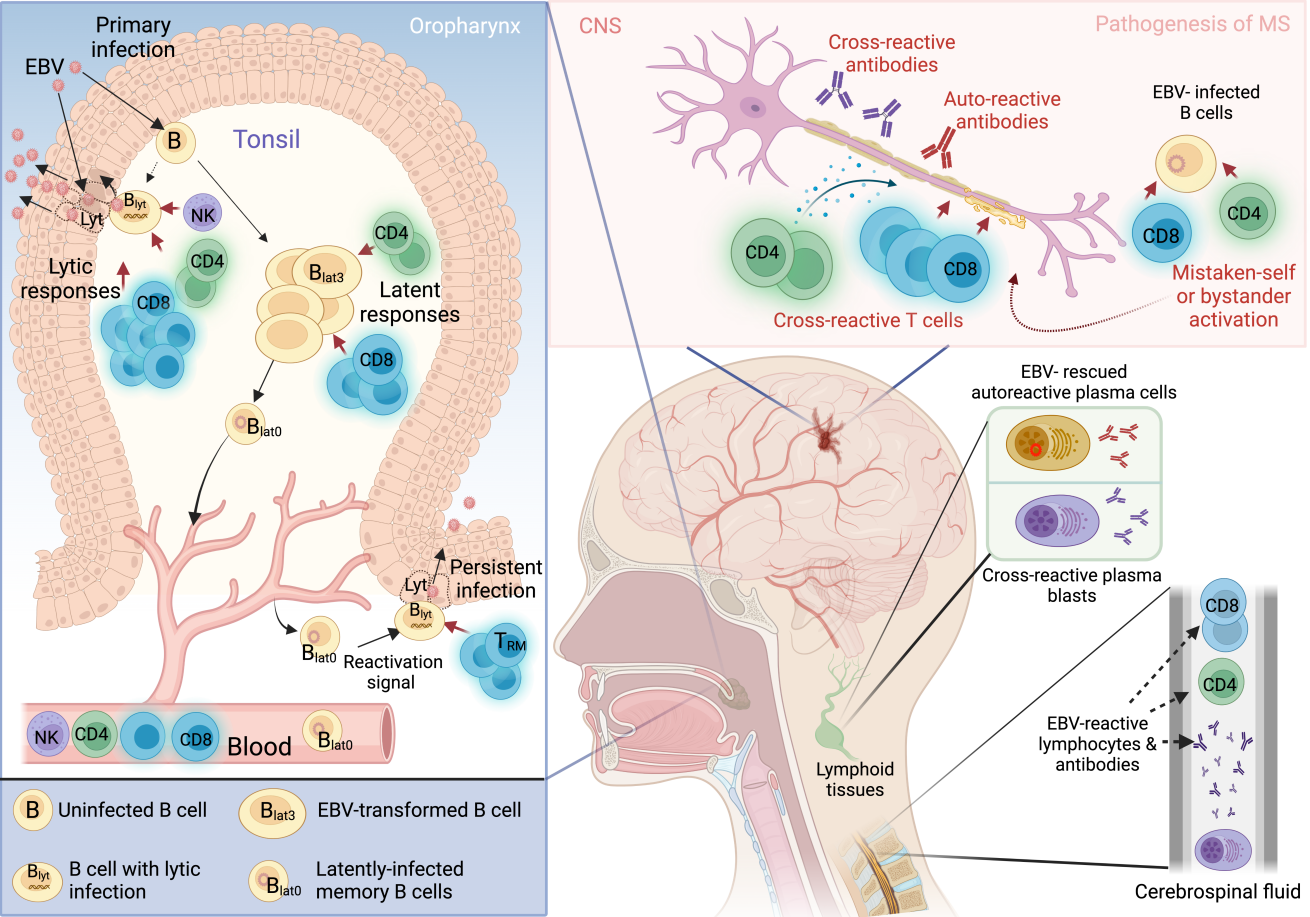
Schematic illustration of the features of EBV infection and the proposed mechanisms implicating its role in MS. Infection spreads through oral secretions and initially EBV replicates locally as a lytic (_lyt_) infection in epithelial cells lining the lymphoid organs of Waldeyer's ring, leading to high levels of viral shedding in the throat. However, early events leading to this initial replication are not fully understood, but there is also evidence to suggest that direct infection of B cells at the surface of these lymphoid organs may proceed epithelial infection.^9^ This lytic infection is gradually brought under control by robust immune responses, the majority of which are EBV‐specific CD8^+^ T cells but also contain both EBV‐specific CD4^+^ T cells and NK cells. Epstein–Barr virus then spreads through initiating a latent growth‐transforming infection of B cells (B_lat3_), resulting in the clonal expansion of the infected pool within oropharyngeal lymphoid tissues. Both CD8^+^ and CD4^+^ T‐cell responses to latent antigens are thought to play key roles in controlling this B cell expansion, but some infected cells appear to escape control through a staged downregulation of latent antigen expression. By the time infected B cells enter the circulation, they have downregulated all latent protein expression (latency 0, B_lat0_), carry the viral DNA as a circular episome, and display a resting (non‐proliferative) memory B‐cell phenotype.^10^ During subsequent long‐term virus carriage (persistent infection), some of the latently infected B cells re‐entering oropharyngeal lymphoid organs may reactivate into lytic cycle, leading to recurrent low‐level shedding of infectious virus into the throat. Such reactivation is likely mainly controlled by tissue‐resident memory CD8^+^ T (T_RM_) cells – and possibly also CD4^+^ T cells^126^ – strategically placed near the epithelial barrier. Right‐most panel: the proposed mechanisms implicating EBV in the pathogenesis of MS are based on some key clinical observations, which include the presence of EBV‐reactive T cells and antibodies in the cerebrospinal fluid and intrathecal maturation of plasma blasts secreting antibodies that cross‐react with CNS proteins. Proposed mechanisms for how EBV may contribute to disease pathogenesis include (1) Rescue of autoreactive B cells. Given EBV's ability to drive proliferation and maturation of B cells independently of T cell help, one potential role for EBV could be that it rescues autoreactive B cells from apoptosis. (2) Molecular mimicry. There is accumulating evidence showing that EBV‐reactive antibodies are also able to recognise self‐antigens via structural homology, leading to production of proinflammatory molecules and resulting in tissue damage. Similarly, there is evidence showing that some EBV‐reactive T cells could also cross‐react with antigens in the CNS. (3) Mistaken self. EBV infection has been found to induce increased expression of self‐proteins on infected B cells, which could in turn be recognised by pathogenic T cells. EBV‐infected B cells have also been identified in the brains of pwMS. It is therefore possible that T cells targeting these cells could cause inflammation and bystander damage to CNS tissue. The figure was created with BioRender.com.

To briefly summarise the currently accepted model of EBV biology, infectious virus is transmitted in saliva and initiates a local, virus‐replicative, ‘lytic’ infection, principally involving epithelial cells lining the lymphoid tissues of the oropharynx; these early events are poorly characterised; however, there is some evidence^9^ to suggest that epithelial cell infection first requires transfer of the virus from locally infiltrating B cells (Figure 1). This oral replication leads to high levels of infectious, viral progeny being detected in throat washings. By that time, based on studies of tonsillar tissues excised from IM patients, EBV has also initiated a growth‐transforming infection of oropharyngeal B cells expressing the full array of latent cycle proteins (referred to as the LCL‐like Latency III programme). Expansions of NK cells and, more dramatically, of CD8^+^ T cells are thought to play a role in containing this lytic infection, though virus replication in the throat is only slowly extinguished. A robust CD8^+^ T‐cell response also targets Latency III‐infected cells, whose numbers fall much more rapidly. However, their decline also reflects the fact that at least some of these cells have already begun a staged downregulation of latent protein expression as they transit to a resting Latency 0 (antigen‐negative, nonproliferative) state (Figure 1)^10^; exactly how this transition occurs is poorly understood. However, as the resultant Latency 0 cells are exclusively found in the circulating IgM^−^IgD^−^ memory B‐cell compartment, analogies have been drawn between latency transition in infected B cells and the physiologic process of memory B‐cell development involving antigen stimulation, clonal proliferation and selection via germinal centre reaction. Once the reservoir of latently infected cells is established, these cells recirculate from the blood through lymphoid organs, preferentially homing to those within the oropharynx, where occasionally reactivation from latent to lytic infection is thought to occur, resulting in renewed low‐level viral shedding in the throat (Figure 1). What triggers such reactivations is again not fully understood; however, as *in vitro* models suggest that the latent to lytic switch can be induced by plasmacytoid differentiation, reactivation *in vivo* may involve chance exposure of memory B cells to cognate antigen, or signals that mimic such antigen‐driven differentiation.^11^ With regard to the carrier state, the available evidence implies a central role for both CD8^+^ and CD4^+^ T cells in long‐term control of EBV infection, with both lytic and latent antigen‐specific memory T‐cell populations easily detectable in the blood of healthy carriers, typically at higher levels than seen against many other viral infections. Epstein–Barr virus also induces antibody responses to both lytic and latent sets of antigens, and these can be useful markers of prior exposure to the virus and in some circumstances of the virus–host balance. Interestingly, such responses (even neutralising antibodies) are slow to develop in acute IM patients, particularly in those with more acute symptoms,^12^ possibly hinting at some contribution of the humoral response to disease resolution. However, the role (if any) of antibodies in stabilising the life‐long asymptomatic carrier state remains unclear.

## Evidence implicating EBV in MS

The first study reporting a link between EBV and MS was published over 40 years ago, when researchers discovered that antibodies against EBV virus capsid antigen (VCA) were significantly elevated in persons with MS than in healthy controls.^13^ Since then, the cumulation of epidemiological and serological evidence has established EBV's role as a likely prerequisite for MS development.

First, studies have shown that > 99% of persons with MS (pwMS) are infected with EBV compared with approximately 95% of the general population.^14^ Multiple prospective studies have consistently reported that EBV infection almost always precedes MS onset, and the largest investigation to date surveyed 10 million individuals showing that risk of disease rises 32‐fold following EBV seroconversion. The same study also showed that individuals who went on to develop MS almost always became EBV seropositive prior to disease onset and that serum levels of neurofilament light chain – a marker of neuronal degeneration – were increased only following EBV infection.^8^ In addition to these observations, it is known that EBV seronegative persons have a vanishingly small MS odds ratio (OR), and crucially this effect is unique to EBV and is not observed for other infections or herpesviruses such as Cytomegalovirus (CMV).^15^ In fact, seropositivity for CMV has been associated with a decreased risk of MS (OR ∼ 0.6),^16^ and one wonders whether this relates to CMV's ability to subtly affect the overall EBV: host balance, as exemplified by the reduced age‐dependent inflation of EBV‐specific T‐cell memory seen in CMV/EBV co‐infected individuals.^17^ Further studies following individuals who went on to develop MS after becoming EBV seropositive also showed that there was a delay between the resolution of primary infection and the development of MS by several months,^7^ suggesting that the subsequent immune dysregulation following primary infection with EBV is driving MS pathogenesis rather than the acute infection itself. Findings of increased EBV seroprevalence are also mirrored in paediatric MS cohorts, where children with disease show significantly increased incidence of EBV carriage than unaffected children.^18^, ^19^, ^20^ Whilst these data indicate that EBV infection is necessary for the development of MS, neurological disease remains rare despite near ubiquitous EBV infection of the general population, suggesting that other environmental or genetic factors are required for disease development.^3^

Second, adaptive immune responses to EBV have been shown by many studies to be altered in pwMS compared with unaffected individuals, the most striking of which are elevated serum antibody titres to EBV nuclear antigens (EBNA). Multiple independent and longitudinal studies have confirmed that antibody responses to EBNA1 and EBNA complex are strongly correlated with risk of MS development, and individuals with the highest antibody titres show up to eight‐ and 36‐fold increased risk of developing disease, respectively.^21^, ^22^ Further studies show that increased risk of MS is specifically linked to antibody responses against the amino acid 385–420 region of EBNA1,^23^, ^24^ that individuals without an EBNA1 antibody response became positive prior to MS onset^7^ and that EBNA1 antibody titres are associated with HLA‐DRB1*15: 01 – the strongest genetic risk factor for MS.^25^, ^26^ EBNA1 antibodies have also been detected in cerebrospinal fluid (CSF);^27^, ^28^ however, these findings were not replicated in other studies.^29^ Serology against further EBV antigens – such as VCA‐specific antibodies – have been reported as elevated in MS, have been detected in CSF and also correlated with loss of brain volume in patients.^30^, ^31^ However, the exact role of inflated antibody responses against EBV antigens in MS development and progression remains somewhat unclear, and the extension of antibody studies to take of advantage of recently developed EBV proteome‐wide assays may be informative in that context.^32^

In addition to increased EBV seroprevalence and perturbed antibody responses in MS, features of virus carriage have been shown to interact with multiple known environmental and genetic disease risk factors. Whilst HLA‐DRB1*15: 01 has been linked to elevated anti‐EBNA IgG titres, no associations have been shown for HLA alleles with history of IM, although one small study showed an effect for HLA‐DRB1*01: 01.^25^, ^26^, ^33^, ^34^, ^35^ Combination of HLA‐DRB1*15: 01 carriage with elevated EBNA1 IgG or clinical history of IM in individuals increases risk of MS 15‐ and sevenfold, respectively.^3^, ^36^ However, whilst these factors all increase risk and together have a multiplicative effect, several studies have shown that these factors act independently.^23^, ^24^, ^37^, ^38^, ^39^ In contrast, HLA‐A*02: 01 is protective (OR ∼ 0.6), and individuals who are HLA‐DRB1*15: 01 positive but are negative for HLA‐A*02: 01 have a combined OR of ∼ 5.^40^, ^41^, ^42^ Epstein–Barr virus infection has also been shown to interact with obesity in adolescence; a BMI of greater than 27 alone has an OR of ∼ 2^43^ but, when combined with EBV seropositivity, this increases 14‐fold.^44^ The reasons for this interaction remain unclear but may be because of the increased proinflammatory milieu associated with obesity, which is known to have a negative impact on the cellular immune response to infections and can induce a state of chronic immune‐mediated inflammation.^45^, ^46^

Third, EBV seroconversion after adolescence carries the highest risk for MS development^47^ but individuals with acute primary infection or those who have recently seroconverted rarely experience neurological symptoms. This significant delay of several months to years following virus acquisition before onset of neurological disease^7^, ^48^ is also consistent with the observation that EBV viral loads in MS patients are mostly unchanged (or only slightly increased) in the peripheral blood of persons with MS compared with healthy controls, indicating that they have already transitioned into a long‐term virus carrier state prior to disease development.^49^, ^50^

## Potential mechanisms implicating EBV in MS

The enigmatic role of the Epstein–Barr virus as a likely prerequisite for MS development has been established; however, the exact neuroinflammatory mechanism through which the effect of the virus is mediated remains unclear. Multiple theories have been put forward for this interaction; however, many are not fully able to explain epidemiological and clinical observations in MS.

One theory suggests that MS is driven by uncontrolled EBV replication in individuals because of decreased T‐cell immunosurveillance and that damage to the CNS is caused by infected B cells gaining access to the CNS. However, debate still surrounds whether cell‐mediated immune responses to EBV are altered in MS, with EBV‐specific T‐cell responses reported as both decreased, unchanged or elevated in pwMS.^27^, ^51^, ^52^, ^53^ Two studies of note reported that CD4^+^ T‐cell responses to EBNA1 were increased in peripheral blood of MS patients and had a Th1 phenotype with a broadened range of epitope specificities compared with healthy controls.^54^, ^55^ Consensus in this field has been hindered in part by high levels of disease heterogeneity in MS, as well by variation in experimental methods; however, mounting evidence suggests that there are subtle differences in the EBV‐specific T‐cell compartment, which may contribute towards MS development and progression.

Research showing a diminished cellular response to EBV has been published for many years;^51^, ^53^ however, this theory is largely incompatible with several key features of disease, such as the observed delay following EBV primary infection before MS onset and the fact that immunosuppressive drugs – such as B‐cell depleting therapies – ameliorate MS disease.^56^ As mentioned above, the delay between primary infection to MS onset has been confirmed by multiple longitudinal studies and is also accompanied by a lack of neurological symptoms in patients with IM.^7^, ^48^ In addition, EBV viral load is elevated in individuals during primary infection, IM and chronically active EBV infection,^57^, ^58^ but multiple studies have shown that this is not the case for MS.^49^, ^50^ Instead, there is a lack of paediatric and adult MS cases or neurological symptoms in individuals with a recent primary infection.^48^ Longitudinal studies have also shown that there is a delay of MS onset following primary infection of at least several months,^7^, ^59^ with a more recent study demonstrating that EBV seroconversion is on average 6.5 years prior to clinical onset.^8^ Incidentally, the appearance of EBNA1‐specific antibodies and CD4^+^ T cells also occurs several months following primary infection.^60^ In addition to this, Natalizumab is a highly effective therapy for MS and blocks VLA‐4‐mediated immune cell trafficking to the CNS and gut which – were uncontrolled replication the mechanism behind MS development – would exacerbate symptoms because of decreased immune surveillance.^61^ Similarly, administration of the antiviral cytokine interferon‐γ in MS patients was found to exacerbate disease.^62^ On the one hand, humanised mice carrying the strongest MS risk allele HLA‐DRB1*15: 01 were shown by one study to have increased EBV load, defective CD4^+^ T‐cell control of EBV‐transformed B‐cell lines and T cells with reactivity for myelin basic protein (MBP) compared with animals carrying an HLA allele not associated with MS.^63^ However, observations from human cohorts intuitively show that HLA‐DRB1*15: 01's role in MS cannot be solely because of defective virus control, as this allele is only associated with MS and not with IM, CAEBV or other diseases that emerge from uncontrolled EBV replication,^25^ and antiviral drug for MS have been shown in clinical trials to be largely ineffective.^64^ Likewise, as HLA class II molecules are also coreceptors for EBV gp42‐mediated fusion of the viral envelope with host cells, one small study showed that viral entry via the HLA‐DRB1*15: 01 was more efficient when compared with another non‐MS‐associated allele.^65^ However, again observations from human cohorts instinctively do not support this observation, as HLA‐DRB1*15: 01 – and particularly homozygosity – is not associated with diseases of uncontrolled replication in humans; however, consequences of increased viral uptake are not fully understood.^25^ The co‐emergence of these phenomena, when coupled with other epidemiological and therapeutic observations, argue against uncontrolled viral replication as the primary mechanism and instead suggests that the immune response to EBV is instead driving pathological processes in MS.

Another theory suggests that immune cell attack on CNS‐infiltrating EBV‐infected B cells causes bystander damage to the CNS and lesion formation. This is supported by some studies that found evidence of EBV‐infected B cells in ectopic lymphoid follicles, lytic replication in active lesions and cytotoxic responses including IFNα production in the brains of postmortem pwMS;^66^, ^67^, ^68^ however, these findings have been contradicted by further investigations.^69^, ^70^, ^71^ Nevertheless, despite their detection in postmortem brain lesions, this does not prove a causal role for EBV‐infected B cells in the brain of pwMS and therefore caution should be exercised when interpreting these findings^72^; although it remains plausible that the presence of EBV‐infected B cells in lesions could exacerbate inflammation through activation of innate immune cells and recruitment of EBV‐specific cytotoxic cells to the area.

Other reports of indirect mechanisms through which EBV could contribute to MS disease pathology have been suggested, such as via activation of human endogenous retroviruses (HERV), and the latent membrane protein LMP2A has been shown to transactivate HERV‐K18 – a superantigen with the ability to activate T cells – in infected B cells.^73^, ^74^ In addition, EBV has been shown to enhance replication of Torque Teno virus, and a further study isolated CD4^+^ T‐cell clones isolated from MS patient CSF with the ability to target multiple epitopes from viruses and autoantigens.^75^, ^76^ Similarly, studies that suggest that EBV may directly mediate damage via infection of CNS‐resident cells have not been corroborated, and there is no strong evidence that EBV is neurotropic.^77^, ^78^, ^79^, ^80^

The capacity of EBV to transform B cells and therefore rescue potential autoreactive B cells from apoptosis has been also suggested as a potential mechanism resulting in neurological disease. Rescued B cells could produce antibodies with affinity for self‐antigens expressed in the brain and gain access to the CNS in an environment with impaired EBV‐specific CD8^+^ T‐cell surveillance. As discussed earlier in this review, reduced cytotoxic T‐cell responses in MS have been previously described in pwMS, and there is also evidence for intrathecal enrichment of EBV‐specific CD8^+^ T cells,^51^, ^53^, ^81^ which could be attributed to infiltration of infected B cells in the CNS. However, analysis of EBV‐infected B cell pools has so far found no evidence for enrichment of autoreactivity.^82^ Other studies have found the EBV‐specific CD8^+^ T‐cell repertoire to be unchanged and even increased in pwMS than in healthy controls,^83^, ^84^ and one study also reported broader EBV‐specific CD8^+^ TCRβ repertoires with central memory phenotype in CSF suggestive of recent antigen priming.^85^

Upregulation of self‐antigens in B cells upon EBV infection which in turn become the target of autoreactive CD4^+^ T‐cell responses has also been reported as a potential mechanism in MS. A key candidate autoantigen for this theory is α‐crystallin B (CRYAB), which has been shown to be upregulated in EBV‐infected B cells *in vitro*,^86^ abundantly expressed in MS brain lesions^87^, ^88^, ^89^ and also a target of CD4^+^ T‐cell responses.^90^ Paradoxically, further studies have shown CRYAB to have a protective and even therapeutic role in neuroinflammation in both the mouse model experimental autoimmune encephalomyelitis (EAE) and MS, with an anti‐apoptotic effect in astrocytes and reduced production of Th1 and Th17 cytokines in T cells.^91^ Most recently, fresh evidence has revealed the existence of cross‐reactive antibodies with the ability to bind homologous epitopes from both CRYAB and EBNA1 in MS, and have also confirmed previous results that CRYAB is a target of autoreactive T‐cell responses in patients.^92^ In line with this theory is the observation that EBV‐transformed B cells elicit cytotoxic CD4^+^ T‐cell responses, which cannot be mapped to any known EBV antigen, indicating that viral transformation may cause aberrant upregulation of self‐antigens, which may become the target of pathogenic T‐cell responses in MS.^93^ Similarly, studies have shown that self‐antigens can be eluted from HLA‐DRB1*15: 01 molecules on the surface of antigen‐presenting cells, which could prime autoproliferative and autoreactive T‐cell responses in MS; however, it is not understood how EBV infection of B cells may influence surface presentation of antigens.^94^, ^95^ Further research is needed to ascertain how EBV infection or viral immune evasion genes influence the immunopeptidome on the B‐cell surface.

Molecular mimicry occurs when adaptive immune responses generated against a foreign pathogen are also able to recognise self‐antigens via structural homology, leading to production of proinflammatory molecules and damage to tissues. Whilst it has been suspected for many years that molecular mimicry with self‐antigens may be driving elevations of EBNA1 IgG in MS, evidence was lacking until recent discoveries showed that antibodies generated against EBNA1 are able to bind homologous epitopes from human proteins, including anoctamin‐2 (ANO2),^96^ glial cell adhesion molecule (GlialCAM)^28^ and CRYAB.^92^ Importantly, these cross‐reactive antibody responses can all be mapped to epitopes within the 380–440 region of EBNA1 – the region to which EBNA1 antibodies also have the highest OR for MS.^23^, ^24^ Evidence that cross‐reactive B cells are primed in the periphery and enter the CNS where they target autoantigens comes from observations that B cells become differentiated in the cervical lymph nodes before their entry into neuronal tissues and that Ig class‐switched memory B cells can traffic between the peripheral sites and the brain.^97^, ^98^ Whilst multiple cross‐reactive or autoreactive antibodies have now been identified in MS, they are only present in a subset of patients and their role in disease has not been clearly defined. It is possible that these antibodies do not directly contribute to disease but instead are biomarkers of pathogenic T‐cell responses which drive disease, as has been shown for other autoimmune disorders such as diabetes mellitus and Addison's disease.^99^, ^100^ Therefore, future work investigating both antibody and T‐cell responses to virus and autoantigens in individuals is of key importance for deciphering role of EBV adaptive immunity in MS.

Wucherpfennig and Strominger first demonstrated that HLA‐DRB1*15: 01‐restricted myelin reactive T‐cell clones from MS patients could recognise homologous peptides from EBV and other viruses,^101^ and since then, several further publications have shown similar cross‐reactivity on the T‐cell level. Lunemann *et al*.^54^ identified a population of expanded EBNA1‐specific CD4^+^ T cells in MS patients, which had broader epitope specificity, were of Th1 central memory or effector memory phenotype and displayed increased reactivity to a pool of myelin antigens. However, no studies to date have isolated single T cells with dual specificity to EBNA1 and self‐proteins, although several examples have been reported for other EBV antigens. Wang *et al*. isolated CD4^+^ T‐cell clones with specificity for RAS guanyl releasing protein 2 (RASGRP2), which were also able to become activated after stimulation with peptides from BPLF1 and BHRF1.^94^ One further study isolated T cells with dual specificity for the EBV DNA polymerase protein BALF5 and MBP epitopes, which were restricted by the MS risk antigen HLA‐DRB1*15: 01 and HLA‐DRB5*01: 01, respectively, from the blood of an MS patient,^102^ and was subsequently found to be expanded in CSF.^103^ However, the current list of T cell autoantigens associated with MS has grown significantly in recent years,^94^, ^104^, ^105^, ^106^ and many questions remain regarding whether T‐cell cross‐recognition of viral and CNS epitopes is a trigger or rather drives progression of disease.

## Missing links and future directions

Being one of the most common infections of humans, EBV has had its fair share of blame for many different diseases, including an association with multiple autoimmune disorders. As with MS, most theories on the role of EBV in other autoimmune diseases – such as rheumatoid arthritis, systemic lupus erythematosus and Sjögren's disease – centre around observations of elevated antibodies to EBV antigens in sera, reduced EBV control and molecular mimicry involving antibodies to EBNA1.^107^, ^108^, ^109^, ^110^, ^111^, ^112^, ^113^ In the case of SLE, disease is somewhat rare in EBV‐negative individuals but IM does not increase risk, and elevated antibodies and viral load can be explained at least in part by nonspecific activation of B cells and defective virus control by cytotoxic T cells.^109^, ^113^ However, there are two clear observations that define EBV's role in MS: (1) this B‐cell‐tropic virus is necessary for the development of MS and (2) B‐cell depletion therapies improve clinical relapse rates in patients, although long‐term data suggest that they do not ultimately prevent disease progression.^56^, ^114^ Also interesting is the rapid effect on relapse rates of anti‐CD20 therapy in patients, which occurs before diminution of B cells has had time to affect the CD20‐negative antibody‐secreting plasma cell compartment. This indicates that clinical efficacy of B‐cell depletion therapies is because of removal of B‐cell functions other than antibody production, such as antigen presentation, production of inflammatory cytokines, stimulation of autoproliferating CD4^+^ T cells, clearing of the EBV‐infected memory B‐cell pool or removal of a subset of proinflammatory CD20^+^ T cells, which have been identified in MS.^105^, ^115^, ^116^, ^117^, ^118^, ^119^

Molecular mimicry is currently the most plausible theory implicating EBV in MS. However, it is also worth noting that there have been inconsistencies on the specificities of the oligoclonal band in CSF between different studies, and only a subset of patients at most have such cross‐reactivity. Importantly, these antibodies are also often polyreactive. In addition, the fact that B‐cell depletion antibodies do not deplete plasma blasts and there is no reduction in oligoclonal bands despite clinical improvement casts some doubt over the role of cross‐reactive antibodies as the primary pathogenic mechanism of EBV in MS, and implicates other B‐cell functions in MS pathogenesis.

Rather than their effector mechanisms, perhaps the pathogenic role of B cells lies in antigen presentation and/or engagement with T cells, and T cell–B cell interactions outside of lymphoid organs are gaining increasing attention.^120^ In this regard, whether EBV‐infected B cells stimulate altered T‐cell responses in MS patients is incompletely understood. The recent study that showed broadening of the EBV‐specific class I‐restricted T‐cell repertoire in pwMS is intriguing but why this happens needs further investigation.^85^ Another study found autoproliferation of Th1 cells driven by memory B cells in an HLA‐DRBR1*15: 01 haplotype‐dependent way in MS patients,^105^ but whether EBV accelerates this process is unknown. Further studies have found higher frequencies of CXCR3^+^ B cells in peripheral blood of pwMS which preferentially infiltrate the CNS of MS patients and are correlated with EBV viral load and EBNA1 antibody titres.^121^ One potential receptor–ligand pair of interest in this context is CD70‐CD27, a costimulatory pathway that leads to up‐regulation of prosurvival genes resulting in the expansion of T cells.^122^ Epstein–Barr virus increases the expression of CD70 on B cells^123^ and, interestingly, increased soluble form of CD27 in CSF is a feature of MS.^124^ Further studies are required to understand the nature of this interaction and how EBV could impact T‐cell responses.

## What can we learn from the link of IM with MS?

The epidemiological evidence showing a greater than twofold increase in MS risk among individuals with a history of IM was one of the foremost drivers of interest in a possible link between EBV and neurological disease. Infectious mononucleosis also shares several epidemiological features with MS: they are both more common in Western countries, their incidence increases with distance from the equator and both are more common in women, in whom they also occur at a slightly younger age.^15^ Whilst post‐IM patients make up only a small fraction of all patients with MS, probably in the region of 15%,^26^ this interaction is an opportunity to ask what it might tell us about MS pathogenesis. Put another way, what are the key differences between symptomatic versus asymptomatic primary EBV infection that might contribute to increased risk of subsequent MS?

There is relatively little published evidence in this area, not least because of challenges in identifying cases of asymptomatic infection, particularly among individuals in the same adolescent/young adult age bracket as IM patients which requires either serendipity or commitment to a huge screening effort. One of the first such studies reported four cases of asymptomatic primary infection identified by chance among volunteers in an early EBV vaccine trial,^125^ whilst a further five cases were identified through large‐scale screening of medical school entrants for EBV and CMV serostatus.^58^ In both studies, detection of IgM antibodies to the EB VCA in healthy subjects was the key identifier; at the time, some of these individuals were still IgG anti‐VCA‐negative, all were IgG anti‐EBNA1‐negative and, where studied prospectively, all showed the much delayed anti‐EBNA1 response typical of IM itself. Importantly, EBV DNA loads were elevated into the same broad range as seen in acute IM patients. However, throughout these studies of asymptomatic infection, there was no evidence of the CD8^+^ T lymphocytosis that characterises the blood picture in IM; in the first study, TCR Vβ screening could not detect obvious T‐cell clonal expansions of the kind seen in IM,^125^ whilst the later work used staining with HLA‐peptide tetramers to show that there were indeed activated EBV‐specific CD8^+^ T cells in the blood but their expansion was much more limited than in acute IM.^58^ Thus, the key difference between symptomatic and asymptomatic primary infection lay not in viral loads, nor it seems (albeit based on relatively crude assays) in the antibody response, but in the size of the virus‐specific CD8^+^ T‐cell response. This forces one to suggest that the overexaggerated T‐cell response may be an important factor underlying IM's role as a risk factor for subsequent MS. Importantly, what drives this exaggerated T‐cell response in IM patients is unclear. Getting to the bottom of this could also shed light onto the possible role of the virus‐specific CD8^+^ T‐cell response in MS. However, given these observations, extreme caution should be exercised when beginning clinical trials of EBV vaccines or EBV‐targeted T‐cell immunotherapies, when we do not fully understand the role of virus‐specific adaptive immune responses in MS pathogenesis.

Perhaps what prompted^6^ to speculate a role for EBV in MS is where the truth lies, and further understanding of IM may be critical in solving the mystery of EBV's role. In that context, amongst those who develop MS, how many have had a delayed, but asymptomatic, primary infection? Put another way, could a high frequency of delayed EBV primary infection – as opposed to a history of symptomatic IM *per se* – underpin the link with MS, and could this explain why MS is becoming increasingly prevalent in developed countries? More generally, is there just one mechanism through which EBV contributes to MS or are several pathways responsible? Genetic analysis of large cohorts in the context of EBV seroconversion – whether symptomatic or not – may also shed light on how virus–host interactions affect the immune compartment at different stages of development. What role do cross‐reactive antibody responses against EBNA1 play, or are they simply biomarkers? How does the growing list of autoantigens associated with MS interact with EBV‐specific adaptive immune responses? We do not fully understand initial triggers or progression of MS – could EBV be solely responsible for the disease trajectory which current therapies cannot ultimately halt? EBV immunology will likely inform future development of personalised MS therapies, but first rigorous studies must be performed to further characterise these virological events in neurological disease.

## Conflict of interest

The authors declare no conflict of interest.

## Author contributions

**Olivia G Thomas:** Writing – original draft; writing – review and editing. **Alan Rickinson:** Writing – original draft; writing – review and editing. **Umaimainthan Palendira:** Writing – original draft; writing – review and editing.

## References

[1] Balfour HHJ , Odumade OA , Schmeling DO *et al*. Behavioral, virologic, and immunologic factors associated with acquisition and severity of primary Epstein‐Barr virus infection in university students. J Infect Dis 2013; 207: 80–88. 23100562 10.1093/infdis/jis646 PMC3523797

[2] Simpson SJ , Wang W , Otahal P , Blizzard L , van der Mei IAF , Taylor BV . Latitude continues to be significantly associated with the prevalence of multiple sclerosis: an updated meta‐analysis. J Neurol Neurosurg Psychiatry 2019; 90: 1193–1200. 31217172 10.1136/jnnp-2018-320189

[3] Olsson T , Barcellos LF , Alfredsson L . Interactions between genetic, lifestyle and environmental risk factors for multiple sclerosis. Nat Rev Neurol 2017; 13: 25–36. 27934854 10.1038/nrneurol.2016.187

[4] Benedikz J , Magnússon H , Guthmundsson G . Multiple sclerosis in Iceland, with observations on the alleged epidemic in The Faroe Islands. Ann Neurol 1994; 36(Suppl 2): S175–S179. 7998786 10.1002/ana.410360804

[5] Binzer S , Imrell K , Binzer M *et al*. Multiple sclerosis in a family on The Faroe Islands. Acta Neurol Scand 2010; 121: 16–19. 19919645 10.1111/j.1600-0404.2009.01291.x

[6] Warner HB , Carp RI . Multiple sclerosis and Epstein‐Barr virus. Lancet 1981; 2: 1290. 10.1016/s0140-6736(81)91527-0 6118702

[7] Levin LI , Munger KL , O'Reilly EJ , Falk KI , Ascherio A . Primary infection with the Epstein‐Barr virus and risk of multiple sclerosis. Ann Neurol 2010; 67: 824–830. 20517945 10.1002/ana.21978 PMC3089959

[8] Bjornevik K , Cortese M , Healy BC *et al*. Longitudinal analysis reveals high prevalence of Epstein‐Barr virus associated with multiple sclerosis. Science 2022; 375: 296–301. 35025605 10.1126/science.abj8222

[9] Dunmire SK , Grimm JM , Schmeling DO , Balfour HHJ , Hogquist KA . The incubation period of primary Epstein‐Barr virus infection: viral dynamics and immunologic events. PLoS Pathog 2015; 11: e1005286. 26624012 10.1371/journal.ppat.1005286 PMC4666617

[10] Laichalk LL , Hochberg D , Babcock GJ , Freeman RB , Thorley‐Lawson DA . The dispersal of mucosal memory B cells: evidence from persistent EBV infection. Immunity 2002; 16: 745–754. 12049725 10.1016/s1074-7613(02)00318-7

[11] Thorley‐Lawson DA , Gross A . Persistence of the Epstein‐Barr virus and the origins of associated lymphomas. N Engl J Med 2004; 350: 1328–1337. 15044644 10.1056/NEJMra032015

[12] Geris JM , Stancari AL , Meirhaeghe MR *et al*. Rapid antibody responses to Epstein‐Barr virus correlate with reduced severity of primary infection. J Clin Virol 2022; 155: 105267. 36007460 10.1016/j.jcv.2022.105267

[13] Sumaya CV , Myers LW , Ellison GW . Epstein‐Barr virus antibodies in multiple sclerosis. Arch Neurol 1980; 37: 94–96. 6243930 10.1001/archneur.1980.00500510052009

[14] Pakpoor J , Disanto G , Gerber JE *et al*. The risk of developing multiple sclerosis in individuals seronegative for Epstein‐Barr virus: a meta‐analysis. Mult Scler 2013; 19: 162–166. 22740437 10.1177/1352458512449682

[15] Ascherio A , Munger KL . Environmental risk factors for multiple sclerosis. Part I: the role of infection. Ann Neurol 2007; 61: 288–299. 17444504 10.1002/ana.21117

[16] Sundqvist E , Bergström T , Daialhosein H *et al*. Cytomegalovirus seropositivity is negatively associated with multiple sclerosis. Mult Scler 2014; 20: 165–173. 23999606 10.1177/1352458513494489

[17] Khan N , Hislop A , Gudgeon N *et al*. Herpesvirus‐specific CD8^+^ T cell immunity in old age: cytomegalovirus impairs the response to a coresident EBV infection. J Immunol 2004; 173: 7481–7489. 15585874 10.4049/jimmunol.173.12.7481

[18] Pohl D , Krone B , Rostasy K *et al*. High seroprevalence of Epstein‐Barr virus in children with multiple sclerosis. Neurology 2006; 67: 2063–2065. 17159123 10.1212/01.wnl.0000247665.94088.8d

[19] Alotaibi S , Kennedy J , Tellier R , Stephens D , Banwell B . Epstein‐Barr virus in pediatric multiple sclerosis. JAMA 2004; 291: 1875–1879. 15100207 10.1001/jama.291.15.1875

[20] Banwell B , Krupp L , Kennedy J *et al*. Clinical features and viral serologies in children with multiple sclerosis: a multinational observational study. Lancet Neurol 2007; 6: 773–781. 17689148 10.1016/S1474-4422(07)70196-5

[21] Munger KL , Levin LI , O'Reilly EJ , Falk KI , Ascherio A . Anti‐Epstein‐Barr virus antibodies as serological markers of multiple sclerosis: a prospective study among United States military personnel. Mult Scler 2011; 17: 1185–1193. 21685232 10.1177/1352458511408991 PMC3179777

[22] Levin LI , Munger KL , Rubertone MV *et al*. Temporal relationship between elevation of epstein‐barr virus antibody titers and initial onset of neurological symptoms in multiple sclerosis. JAMA 2005; 293: 2496–2500. 15914750 10.1001/jama.293.20.2496

[23] Sundström P , Nyström M , Ruuth K , Lundgren E . Antibodies to specific EBNA‐1 domains and HLA DRB1*1501 interact as risk factors for multiple sclerosis. J Neuroimmunol 2009; 215: 102–107. 19733917 10.1016/j.jneuroim.2009.08.004

[24] Sundqvist E , Sundström P , Lindén M *et al*. Epstein‐Barr virus and multiple sclerosis: interaction with HLA. Genes Immun 2012; 13: 14–20. 21776012 10.1038/gene.2011.42

[25] Huang J. Biomarkers and viral risk factors in multiple sclerosis. Doctoral Thesis, Karolinska Institutet, Sweden; 2022.

[26] Hedström AK , Huang J , Michel A *et al*. High levels of Epstein‐Barr virus nuclear Antigen‐1‐specific antibodies and infectious mononucleosis act both independently and synergistically to increase multiple sclerosis risk. Front Neurol 2019; 10: 1368. 32038456 10.3389/fneur.2019.01368 PMC6992610

[27] Cepok S , Zhou D , Srivastava R *et al*. Identification of Epstein‐Barr virus proteins as putative targets of the immune response in multiple sclerosis. J Clin Invest 2005; 115: 1352–1360. 15841210 10.1172/JCI23661 PMC1077174

[28] Lanz TV , Brewer RC , Ho PP *et al*. Clonally expanded B cells in multiple sclerosis bind EBV EBNA1 and GlialCAM. Nature 2022; 603: 321–327. 35073561 10.1038/s41586-022-04432-7 PMC9382663

[29] Jafari N , van Nierop GP , Verjans GMGM , Osterhaus ADME , Middeldorp JM , Hintzen RQ . No evidence for intrathecal IgG synthesis to Epstein Barr virus nuclear antigen‐1 in multiple sclerosis. J Clin Virol 2010; 49: 26–31 do7. 20638898 10.1016/j.jcv.2010.06.007

[30] Zivadinov R , Zorzon M , Weinstock‐Guttman B *et al*. Epstein‐Barr virus is associated with grey matter atrophy in multiple sclerosis. J Neurol Neurosurg Psychiatry 2009; 80: 620–625. 19168469 10.1136/jnnp.2008.154906

[31] Castellazzi M , Tamborino C , Cani A *et al*. Epstein‐Barr virus‐specific antibody response in cerebrospinal fluid and serum of patients with multiple sclerosis. Mult Scler 2010; 16: 883–887. 20483883 10.1177/1352458510368051

[32] Argirion I , Pfeiffer RM , Proietti C *et al*. Comparative analysis of the humoral immune response to the EBV proteome across EBV‐related malignancies. Cancer Epidemiol Biomarkers Prev 2023; 32: 687–696 Published online February 14, EPI‐22‐0452. 36788424 10.1158/1055-9965.EPI-22-0452 PMC10159936

[33] Ramagopalan SV , Meier UC , Conacher M *et al*. Role of the HLA system in the association between multiple sclerosis and infectious mononucleosis. Arch Neurol 2011; 68: 469–472. 21482926 10.1001/archneurol.2011.48

[34] Waubant E , Mowry EM , Krupp L *et al*. Antibody response to common viruses and human leukocyte antigen‐DRB1 in pediatric multiple sclerosis. Mult Scler 2013; 19: 891–895. 23232601 10.1177/1352458512469693 PMC3665694

[35] Rubicz R , Yolken R , Drigalenko E *et al*. A genome‐wide integrative genomic study localizes genetic factors influencing antibodies against Epstein‐Barr virus nuclear antigen 1 (EBNA‐1). PLoS Genet 2013; 9: e1003147. 23326239 10.1371/journal.pgen.1003147 PMC3542101

[36] Nielsen TR , Rostgaard K , Askling J *et al*. Effects of infectious mononucleosis and HLA‐DRB1*15 in multiple sclerosis. Mult Scler 2009; 15: 431–436. 19153174 10.1177/1352458508100037

[37] De Jager PL , Simon KC , Munger KL , Rioux JD , Hafler DA , Ascherio A . Integrating risk factors: HLA‐DRB1*1501 and Epstein‐Barr virus in multiple sclerosis. Neurology 2008; 70(13 Pt 2): 1113–1118. 18272866 10.1212/01.wnl.0000294325.63006.f8

[38] Disanto G , Hall C , Lucas R *et al*. Assessing interactions between HLA‐DRB1*15 and infectious mononucleosis on the risk of multiple sclerosis. Mult Scler 2013; 19: 1355–1358. 23413297 10.1177/1352458513477231

[39] Simon KC , Schmidt H , Loud S , Ascherio A . Risk factors for multiple sclerosis, neuromyelitis optica and transverse myelitis. Mult Scler 2015; 21: 703–709. 25305254 10.1177/1352458514551780

[40] Beecham AH , Patsopoulos NA , Xifara DK *et al*. Analysis of immune‐related loci identifies 48 new susceptibility variants for multiple sclerosis. Nat Genet 2013; 45: 1353–1360. 24076602 10.1038/ng.2770 PMC3832895

[41] Brynedal B , Duvefelt K , Jonasdottir G *et al*. HLA‐A confers an HLA‐DRB1 independent influence on the risk of multiple sclerosis. PLoS One 2007; 2: e664. 17653284 10.1371/journal.pone.0000664 PMC1919434

[42] Sawcer S , Hellenthal G , Pirinen M *et al*. Genetic risk and a primary role for cell‐mediated immune mechanisms in multiple sclerosis. Nature 2011; 476: 214–219. 21833088 10.1038/nature10251 PMC3182531

[43] Hedström AK , Lima Bomfim I , Barcellos L *et al*. Interaction between adolescent obesity and HLA risk genes in the etiology of multiple sclerosis. Neurology 2014; 82: 865–872. 24500647 10.1212/WNL.0000000000000203 PMC3959752

[44] Hedström AK , Lima Bomfim I , Hillert J , Olsson T , Alfredsson L . Obesity interacts with infectious mononucleosis in risk of multiple sclerosis. Eur J Neurol 2015; 22: 578–e38. 25530445 10.1111/ene.12620 PMC4365756

[45] Karlsson EA , Beck MA . The burden of obesity on infectious disease. Exp Biol Med (Maywood) 2010; 235: 1412–1424. 21127339 10.1258/ebm.2010.010227

[46] Subramanian V , Ferrante AWJ . Obesity, inflammation, and macrophages. Nestle Nutr Workshop Ser Pediatr Program 2009; 63: 151–159 discussion 159–62, 259–268. 10.1159/000209979 19346774

[47] Biström M , Jons D , Engdahl E *et al*. Epstein‐Barr virus infection after adolescence and human herpesvirus 6A as risk factors for multiple sclerosis. Eur J Neurol 2021; 28: 579–586. 33065762 10.1111/ene.14597 PMC7839468

[48] Yea C , Tellier R , Chong P *et al*. Epstein‐Barr virus in oral shedding of children with multiple sclerosis. Neurology 2013; 81: 1392–1399. 24014504 10.1212/WNL.0b013e3182a841e4 PMC3806908

[49] Wagner HJ , Munger KL , Ascherio A . Plasma viral load of Epstein‐Barr virus and risk of multiple sclerosis. Eur J Neurol 2004; 11: 833–834. 15667414 10.1111/j.1468-1331.2004.00871.x

[50] Lindsey JW , Hatfield LM , Crawford MP , Patel S . Quantitative PCR for Epstein‐Barr virus DNA and RNA in multiple sclerosis. Mult Scler 2009; 15: 153–158. 18845656 10.1177/1352458508097920

[51] Jilek S , Schluep M , Harari A *et al*. HLA‐B7‐restricted EBV‐specific CD8^+^ T cells are dysregulated in multiple sclerosis. J Immunol 2012; 188: 4671–4680. 22461701 10.4049/jimmunol.1103100

[52] Höllsberg P , Hansen HJ , Haahr S . Altered CD8^+^ T cell responses to selected Epstein‐Barr virus immunodominant epitopes in patients with multiple sclerosis. Clin Exp Immunol 2003; 132: 137–143. 12653848 10.1046/j.1365-2249.2003.02114.x PMC1808679

[53] Pender MP , Csurhes PA , Burrows JM , Burrows SR . Defective T‐cell control of Epstein‐Barr virus infection in multiple sclerosis. Clin Transl Immunology 2017; 6: e126. 28197337 10.1038/cti.2016.87 PMC5292561

[54] Lünemann JD , Jelcić I , Roberts S *et al*. EBNA1‐specific T cells from patients with multiple sclerosis cross react with myelin antigens and co‐produce IFN‐γ and IL‐2. J Exp Med 2008; 205: 1763–1773. 18663124 10.1084/jem.20072397 PMC2525578

[55] Lünemann JD , Edwards N , Muraro PA *et al*. Increased frequency and broadened specificity of latent EBV nuclear antigen‐1‐specific T cells in multiple sclerosis. Brain 2006; 129(Pt 6): 1493–1506. 16569670 10.1093/brain/awl067

[56] Salzer J , Svenningsson R , Alping P *et al*. Rituximab in multiple sclerosis: a retrospective observational study on safety and efficacy. Neurology 2016; 87: 2074–2081. 27760868 10.1212/WNL.0000000000003331 PMC5109942

[57] Kimura H , Hoshino Y , Kanegane H *et al*. Clinical and virologic characteristics of chronic active Epstein‐Barr virus infection. Blood 2001; 98: 280–286. 11435294 10.1182/blood.v98.2.280

[58] Abbott RJ , Pachnio A , Pedroza‐Pacheco I *et al*. Asymptomatic primary infection with Epstein‐Barr virus: observations on young adult cases. J Virol 2017; 91: e00382‐17. 28835490 10.1128/JVI.00382-17 PMC5640854

[59] Endriz J , Ho PP , Steinman L . Time correlation between mononucleosis and initial symptoms of MS. Neurol Neuroimmunol Neuroinflammation 2017; 4: e308. 10.1212/NXI.0000000000000308 PMC5330199 28271078

[60] Long HM , Chagoury OL , Leese AM *et al*. MHC II tetramers visualize human CD4^+^ T cell responses to Epstein‐Barr virus infection and demonstrate atypical kinetics of the nuclear antigen EBNA1 response. J Exp Med 2013; 210: 933–949. 23569328 10.1084/jem.20121437 PMC3646497

[61] Polman CH , O'Connor PW , Havrdova E *et al*. A randomized, placebo‐controlled trial of natalizumab for relapsing multiple sclerosis. N Engl J Med 2006; 354: 899–910 219: e20220650. 16510744 10.1056/NEJMoa044397

[62] Panitch HS , Hirsch RL , Haley AS , Johnson KP . Exacerbations of multiple sclerosis in patients treated with γ interferon. Lancet 1987; 1: 893–895. 2882294 10.1016/s0140-6736(87)92863-7

[63] Zdimerova H , Murer A , Engelmann C *et al*. Attenuated immune control of Epstein‐Barr virus in humanized mice is associated with the multiple sclerosis risk factor HLA‐DR15. Eur J Immunol 2021; 51: 64–75. 32949466 10.1002/eji.202048655

[64] Lycke J . Trials of antivirals in the treatment of multiple sclerosis. Acta Neurol Scand 2017; 136(Suppl 201): 45–48. 29068492 10.1111/ane.12839

[65] Menegatti J , Schub D , Schäfer M , Grässer FA , Ruprecht K . HLA‐DRB1*15: 01 is a co‐receptor for Epstein‐Barr virus, linking genetic and environmental risk factors for multiple sclerosis. Eur J Immunol 2021; 51: 2348–2350. 34019695 10.1002/eji.202149179

[66] Serafini B , Rosicarelli B , Franciotta D *et al*. Dysregulated Epstein‐Barr virus infection in the multiple sclerosis brain. J Exp Med 2007; 204: 2899–2912. 17984305 10.1084/jem.20071030 PMC2118531

[67] Tzartos JS , Khan G , Vossenkamper A *et al*. Association of innate immune activation with latent Epstein‐Barr virus in active MS lesions. Neurology 2012; 78: 15–23. 22156987 10.1212/WNL.0b013e31823ed057

[68] Serafini B , Scorsi E , Rosicarelli B , Rigau V , Thouvenot E , Aloisi F . Massive intracerebral Epstein‐Barr virus reactivation in lethal multiple sclerosis relapse after natalizumab withdrawal. J Neuroimmunol 2017; 307: 14–17. 28495131 10.1016/j.jneuroim.2017.03.013

[69] Peferoen LAN , Lamers F , Lodder LNR *et al*. Epstein Barr virus is not a characteristic feature in the central nervous system in established multiple sclerosis. Brain 2010; 133(Pt 5): e137. 19917644 10.1093/brain/awp296

[70] Willis SN , Stadelmann C , Rodig SJ *et al*. Epstein‐Barr virus infection is not a characteristic feature of multiple sclerosis brain. Brain 2009; 132(Pt 12): 3318–3328. 19638446 10.1093/brain/awp200 PMC2792367

[71] Sargsyan SA , Shearer AJ , Ritchie AM *et al*. Absence of Epstein‐Barr virus in the brain and CSF of patients with multiple sclerosis. Neurology 2010; 74: 1127–1135. 20220124 10.1212/WNL.0b013e3181d865a1 PMC2865779

[72] Lassmann H , Niedobitek G , Aloisi F , Middeldorp JM . Epstein‐Barr virus in the multiple sclerosis brain: a controversial issue – report on a focused workshop held in the Centre for Brain Research of the Medical University of Vienna. Austria Brain 2011; 134(Pt 9): 2772–2786. 21846731 10.1093/brain/awr197 PMC3170536

[73] Tai AK , O'Reilly EJ , Alroy KA *et al*. Human endogenous retrovirus‐K18 env as a risk factor in multiple sclerosis. Mult Scler 2008; 14: 1175–1180. 18701576 10.1177/1352458508094641 PMC2754175

[74] Hsiao FC , Tai AK , Deglon A , Sutkowski N , Longnecker R , Huber BT . EBV LMP‐2A employs a novel mechanism to transactivate the HERV‐K18 superantigen through its ITAM. Virology 2009; 385: 261–266. 19070345 10.1016/j.virol.2008.11.025

[75] Borkosky SS , Whitley C , Kopp‐Schneider A , zur Hausen H , de Villiers EM . Epstein‐Barr virus stimulates torque Teno virus replication: a possible relationship to multiple sclerosis. PLoS One 2012; 7: e32160. 22384166 10.1371/journal.pone.0032160 PMC3285200

[76] Sospedra M , Zhao Y , zur Hausen H *et al*. Recognition of conserved amino acid motifs of common viruses and its role in autoimmunity. PLoS Pathog 2005; 1: e41. 16362076 10.1371/journal.ppat.0010041 PMC1315278

[77] Menet A , Speth C , Larcher C *et al*. Epstein‐Barr virus infection of human astrocyte cell lines. J Virol 1999; 73: 7722–7733. 10438862 10.1128/jvi.73.9.7722-7733.1999 PMC104299

[78] Jha HC , Mehta D , Lu J *et al*. γherpesvirus infection of human neuronal cells. MBio 2015; 6: e01844‐15. 26628726 10.1128/mBio.01844-15 PMC4669387

[79] Jakhmola S , Jha HC . Glial cell response to Epstein‐Barr virus infection: a plausible contribution to virus‐associated inflammatory reactions in the brain. Virology 2021; 559: 182–195. 33964684 10.1016/j.virol.2021.04.005

[80] Hassani A , Corboy JR , Al‐Salam S , Khan G . Epstein‐Barr virus is present in the brain of most cases of multiple sclerosis and may engage more than just B cells. PLoS One 2018; 13: e0192109. 29394264 10.1371/journal.pone.0192109 PMC5796799

[81] Jaquiéry E , Jilek S , Schluep M *et al*. Intrathecal immune responses to EBV in early MS. Eur J Immunol 2010; 40: 878–887. 20017197 10.1002/eji.200939761

[82] Tracy SI , Kakalacheva K , Lünemann JD , Luzuriaga K , Middeldorp J , Thorley‐Lawson DA . Persistence of Epstein‐Barr virus in self‐reactive memory B cells. J Virol 2012; 86: 12330–12340. 22951828 10.1128/JVI.01699-12 PMC3486485

[83] Jilek S , Schluep M , Meylan P *et al*. Strong EBV‐specific CD8^+^ T‐cell response in patients with early multiple sclerosis. Brain 2008; 131(Pt 7): 1712–1721. 18550621 10.1093/brain/awn108

[84] Lindsey JW , Hatfield LM . Epstein‐Barr virus and multiple sclerosis: cellular immune response and cross‐reactivity. J Neuroimmunol 2010; 229: 238–242. 20826010 10.1016/j.jneuroim.2010.08.009

[85] Schneider‐Hohendorf T , Gerdes LA , Pignolet B *et al*. Broader Epstein‐Barr virus‐specific T cell receptor repertoire in patients with multiple sclerosis. J Exp Med 2022; 219: e20220650. 36048016 10.1084/jem.20220650 PMC9437111

[86] van Sechel AC , Bajramovic JJ , van Stipdonk MJ , Persoon‐Deen C , Geutskens SB , van Noort JM . EBV‐induced expression and HLA‐DR‐restricted presentation by human B cells of αB‐crystallin, a candidate autoantigen in multiple sclerosis. J Immunol 1999; 162: 129–135. 9886378

[87] Bajramović JJ , Lassmann H , van Noort JM . Expression of αB‐crystallin in glia cells during lesional development in multiple sclerosis. J Neuroimmunol 1997; 78: 143–151. 9307239 10.1016/s0165-5728(97)00092-1

[88] van Noort JM , van Sechel AC , Bajramovic JJ *et al*. The small heat‐shock protein αB‐crystallin as candidate autoantigen in multiple sclerosis. Nature 1995; 375: 798–801. 7596414 10.1038/375798a0

[89] van Noort JM , Bsibsi M , Gerritsen WH *et al*. αB‐crystallin is a target for adaptive immune responses and a trigger of innate responses in preactive multiple sclerosis lesions. J Neuropathol Exp Neurol 2010; 69: 694–703. 20535035 10.1097/NEN.0b013e3181e4939c

[90] Chou YK , Burrows GG , LaTocha D *et al*. CD4^+^ T‐cell epitopes of human αB‐crystallin. J Neurosci Res 2004; 75: 516–523. 14743435 10.1002/jnr.20000

[91] Ousman SS , Tomooka BH , van Noort JM *et al*. Protective and therapeutic role for αB‐crystallin in autoimmune demyelination. Nature 2007; 448: 474–479. 17568699 10.1038/nature05935

[92] Thomas OG , Bronge M , Tengvall K *et al*. Cross‐reactive EBNA1 immunity targets α‐crystallin B and is associated with multiple sclerosis. Sci Adv 2023; 9. 10.1126/sciadv.adg3032 PMC10191428 37196088

[93] Long HM , Zuo J , Leese AM *et al*. CD4^+^ T‐cell clones recognizing human lymphoma‐associated antigens: generation by *in vitro* stimulation with autologous Epstein‐Barr virus‐transformed B cells. Blood 2009; 114: 807–815. 19443664 10.1182/blood-2008-12-194043

[94] Wang J , Jelcic I , Mühlenbruch L *et al*. HLA‐DR15 molecules jointly shape an autoreactive T cell repertoire in multiple sclerosis. Cell 2020; 183: 1264–1281.e20. 33091337 10.1016/j.cell.2020.09.054 PMC7707104

[95] Mohme M , Hotz C , Stevanovic S *et al*. HLA‐DR15‐derived self‐peptides are involved in increased autologous T cell proliferation in multiple sclerosis. Brain 2013; 136(Pt 6): 1783–1798. 23739916 10.1093/brain/awt108

[96] Tengvall K , Huang J , Hellström C *et al*. Molecular mimicry between anoctamin 2 and Epstein‐Barr virus nuclear antigen 1 associates with multiple sclerosis risk. Proc Natl Acad Sci USA 2019; 116: 6. 10.1073/pnas.1902623116 PMC6708327 31375628

[97] Stern JNH , Yaari G , Vander Heiden JA *et al*. B cells populating the multiple sclerosis brain mature in the draining cervical lymph nodes. Sci Transl Med 2014; 6: 248ra107. 10.1126/scitranslmed.3008879 PMC4388137 25100741

[98] Palanichamy A , Apeltsin L , Kuo TC *et al*. Immunoglobulin class‐switched B cells form an active immune axis between CNS and periphery in multiple sclerosis. Sci Transl Med 2014; 6: 248ra106. 10.1126/scitranslmed.3008930 PMC4176763 25100740

[99] Pugliese A . Autoreactive T cells in type 1 diabetes. J Clin Invest 2017; 127: 2881–2891. 28762987 10.1172/JCI94549 PMC5531393

[100] Dawoodji A , Chen JL , Shepherd D *et al*. High frequency of cytolytic 21‐hydroxylase‐specific CD8^+^ T cells in autoimmune Addison's disease patients. J Immunol 2014; 193: 2118–2126. 25063864 10.4049/jimmunol.1400056 PMC4821366

[101] Wucherpfennig KW , Strominger JL . Molecular mimicry in T cell‐mediated autoimmunity: viral peptides activate human T cell clones specific for myelin basic protein. Cell 1995; 80: 695–705. 7534214 10.1016/0092-8674(95)90348-8 PMC7133435

[102] Lang HLE , Jacobsen H , Ikemizu S *et al*. A functional and structural basis for TCR cross‐reactivity in multiple sclerosis. Nat Immunol 2002; 3: 940–943. 12244309 10.1038/ni835

[103] Holmøy T , Kvale EØ , Vartdal F . Cerebrospinal fluid CD4^+^ T cells from a multiple sclerosis patient cross‐recognize Epstein‐Barr virus and myelin basic protein. J Neurovirol 2004; 10: 278–283. 15385250 10.1080/13550280490499524

[104] Bronge M , Högelin KA , Thomas OG *et al*. Identification of four novel T cell autoantigens and personal autoreactive profiles in multiple sclerosis. Sci Adv 2022; 8: eabn1823. 35476434 10.1126/sciadv.abn1823 PMC9045615

[105] Jelcic I , Al Nimer F , Wang J *et al*. Memory B cells activate brain‐homing, autoreactive CD4^+^ T cells in multiple sclerosis. Cell 2018; 175: 85–100.e23. 30173916 10.1016/j.cell.2018.08.011 PMC6191934

[106] Planas R , Santos R , Tomas‐Ojer P *et al*. GDP‐l‐fucose synthase is a CD4^+^ T cell‐specific autoantigen in DRB3*02: 02 patients with multiple sclerosis. Sci Transl Med 2018; 10: eaat4301. 30305453 10.1126/scitranslmed.aat4301

[107] McClain MT , Rapp EC , Harley JB , James JA . Infectious mononucleosis patients temporarily recognize a unique, cross‐reactive epitope of Epstein‐Barr virus nuclear antigen‐1. J Med Virol 2003; 70: 5. 10.1002/jmv.10385 12696112

[108] McClain MT , Heinlen LD , Dennis GJ , Roebuck J , Harley JB , James JA . Early events in lupus humoral autoimmunity suggest initiation through molecular mimicry. Nat Med 2005; 11: 85–89. 15619631 10.1038/nm1167

[109] Hanlon P , Avenell A , Aucott L , Vickers MA . Systematic review and meta‐analysis of the sero‐epidemiological association between Epstein‐Barr virus and systemic lupus erythematosus. Arthritis Res Ther 2014; 16: R3. 24387619 10.1186/ar4429 PMC3978841

[110] Tsokos GC . Systemic lupus erythematosus. N Engl J Med 2011; 365: 2110–2121. 22129255 10.1056/NEJMra1100359

[111] James JA , Kaufman KM , Farris AD , Taylor‐Albert E , Lehman TJ , Harley JB . An increased prevalence of Epstein‐Barr virus infection in young patients suggests a possible etiology for systemic lupus erythematosus. J Clin Invest 1997; 100: 6. 9399948 10.1172/JCI119856 PMC508514

[112] Liu Z , Chu A . Sjögren's syndrome and viral infections. Rheumatol Ther 2021; 8: 1051–1059. 34227038 10.1007/s40744-021-00334-8 PMC8380615

[113] Kang I , Quan T , Nolasco H *et al*. Defective control of latent Epstein‐Barr virus infection in systemic lupus erythematosus. J Immunol 2004; 172: 1287–1294. 14707107 10.4049/jimmunol.172.2.1287

[114] Hawker K , O'Connor P , Freedman MS *et al*. Rituximab in patients with primary progressive multiple sclerosis: results of a randomized double‐blind placebo‐controlled multicenter trial. Ann Neurol 2009; 66: 460–471. 19847908 10.1002/ana.21867

[115] Palanichamy A , Jahn S , Nickles D *et al*. Rituximab efficiently depletes increased CD20‐expressing T cells in multiple sclerosis patients. J Immunol 2014; 193: 580–586. 24928997 10.4049/jimmunol.1400118 PMC4082756

[116] Lassmann H . Pathogenic mechanisms associated with different clinical courses of multiple sclerosis. Front Immunol 2018; 9: 3116. 30687321 10.3389/fimmu.2018.03116 PMC6335289

[117] Hauser SL , Waubant E , Arnold DL *et al*. B‐cell depletion with rituximab in relapsing‐remitting multiple sclerosis. N Engl J Med 2008; 358: 676–688. 18272891 10.1056/NEJMoa0706383

[118] Lisak RP , Benjamins JA , Nedelkoska L *et al*. Secretory products of multiple sclerosis B cells are cytotoxic to oligodendroglia *in vitro* . J Neuroimmunol 2012; 246: 85–95. 22458983 10.1016/j.jneuroim.2012.02.015

[119] von Büdingen HC , Bar‐Or A , Zamvil SS . B cells in multiple sclerosis: connecting the dots. Curr Opin Immunol 2011; 23: 713–720. 21983151 10.1016/j.coi.2011.09.003 PMC4188435

[120] Naderi W , Schreiner D , King CG . T‐cell–B‐cell collaboration in the lung. Curr Opin Immunol 2023; 81: 102284. 36753826 10.1016/j.coi.2023.102284

[121] van Langelaar J , Wierenga‐Wolf AF , Samijn JPA *et al*. The association of Epstein‐Barr virus infection with CXCR3^+^ B‐cell development in multiple sclerosis: impact of immunotherapies. Eur J Immunol 2021; 51: 626–633. 33152118 10.1002/eji.202048739 PMC7984177

[122] Song DG , Ye Q , Poussin M , Harms GM , Figini M , Powell DJ Jr . CD27 costimulation augments the survival and antitumor activity of redirected human T cells *in vivo* . Blood 2012; 119: 696–706. 22117050 10.1182/blood-2011-03-344275

[123] Izawa K , Martin E , Soudais C *et al*. Inherited CD70 deficiency in humans reveals a critical role for the CD70‐CD27 pathway in immunity to Epstein‐Barr virus infection. J Exp Med 2017; 214: 73–89. 28011863 10.1084/jem.20160784 PMC5206497

[124] Hintzen RQ , van Lier RW , Kuijpers KC *et al*. Elevated levels of a soluble form of the T cell activation antigen CD27 in cerebrospinal fluid of multiple sclerosis patients. J Neuroimmunol 1991; 35: 211–217. 1659587 10.1016/0165-5728(91)90175-7

[125] Silins SL , Sherritt MA , Silleri JM *et al*. Asymptomatic primary Epstein‐Barr virus infection occurs in the absence of blood T‐cell repertoire perturbations despite high levels of systemic viral load. Blood 2001; 98: 3739–3744. 11739180 10.1182/blood.v98.13.3739

[126] Niessl J , Sekine T , Lange J *et al*. Identification of resident memory CD8^+^ T cells with functional specificity for SARS‐CoV‐2 in unexposed oropharyngeal lymphoid tissue. Sci Immunol 2021; 6: eabk0894. 34519539 10.1126/sciimmunol.abk0894 PMC10763663

